# Wooden Plate Denture Reproduced Using Materials and Methods From 400 Years Ago

**DOI:** 10.7759/cureus.75641

**Published:** 2024-12-13

**Authors:** Kazuya Yoshida

**Affiliations:** 1 Department of Oral and Maxillofacial Surgery, National Hospital Organization, Kyoto Medical Center, Kyoto, JPN

**Keywords:** boxwood, complete denture, edo period, history, masticatory performance, occlusal force, reproduction, suction retention, wooden plate denture

## Abstract

This study aimed to reproduce a complete wooden plate denture, which was the first in the world to retain suction under negative pressure, using the same materials and methods from 400 years ago (i.e., the Edo period) to verify its masticatory performance. A complete wooden plate denture was fabricated based on the maxilla of a 90-year-old female. An impression of the maxilla was made using beeswax, which was pressed onto the negative impression to create a working model. Red food coloring was applied to the working model and pressed onto a wooden denture base made of boxwood, and the marked areas were then carved with chisels and carving knives. Food coloring was applied to the alveolar ridges of the subject and the fit was adjusted. Ivory was used to create artificial teeth, which were carved in a dovetail form and embedded in Japanese raw lacquer. Masticatory performance was assessed using the gummy-jelly test and occlusal force measurement, and the results of current and wooden dentures were compared. The masticatory ability of the wooden denture (185 mg/dL) was lower than that of the current denture (209 mg/dl). In contrast, the mean occlusal force was slightly higher with the wooden denture (31.8 MPa) than with the current denture (31.4 MPa). This study suggests that wooden plate dentures were likely functional during the period when they were used. Japanese wooden dentures hold significant importance in the evolution of denture technology.

## Introduction

In Japan, highly sophisticated wood-processing techniques have been developed in various fields such as Buddhist statues, architecture, furniture, Noh masks, and "netsukes" (miniature carved Japanese sculptures, typically made of wood, ivory, or bone, often used as a toggle to fasten a cord to a kimono). The modern theory of complete denture retention using suction was applied to wooden plate dentures in Japan from the first half of the 16th century [1, 2], which is an astonishing achievement since it was clinically applied in North America and Europe in the latter half of the 19th century nearly 300 years later [3]. Japanese wooden plate dentures have not been mentioned in Western literature on the history of dentistry, except in the writing of Hoffmann-Axthelm [4]. As a result, these historical facts are rarely recognized outside Japan.

This study aimed to reproduce a complete Japanese wooden plate denture using the same materials, tools, and methods used 400 years ago and to verify its masticatory performance.

## Technical report

Patient characteristics, materials, and methods

The participant was a healthy 90-year-old female whose maxilla was edentulous and who wore a complete denture. Her left lower second premolar and the first and second molars were missing, leaving only the first molar, and she wore a removable partial denture. This study was conducted in accordance with the guidelines of the Declaration of Helsinki and approved by the Institutional Review Board and Ethics Committee of the Kyoto Medical Center. The patient received an explanation of the treatment plan and provided written informed consent. The materials, tools, and techniques used to fabricate the wooden plate denture were based on previous studies [1-3, 5].

Fabrication of the denture

**Figure 1:**
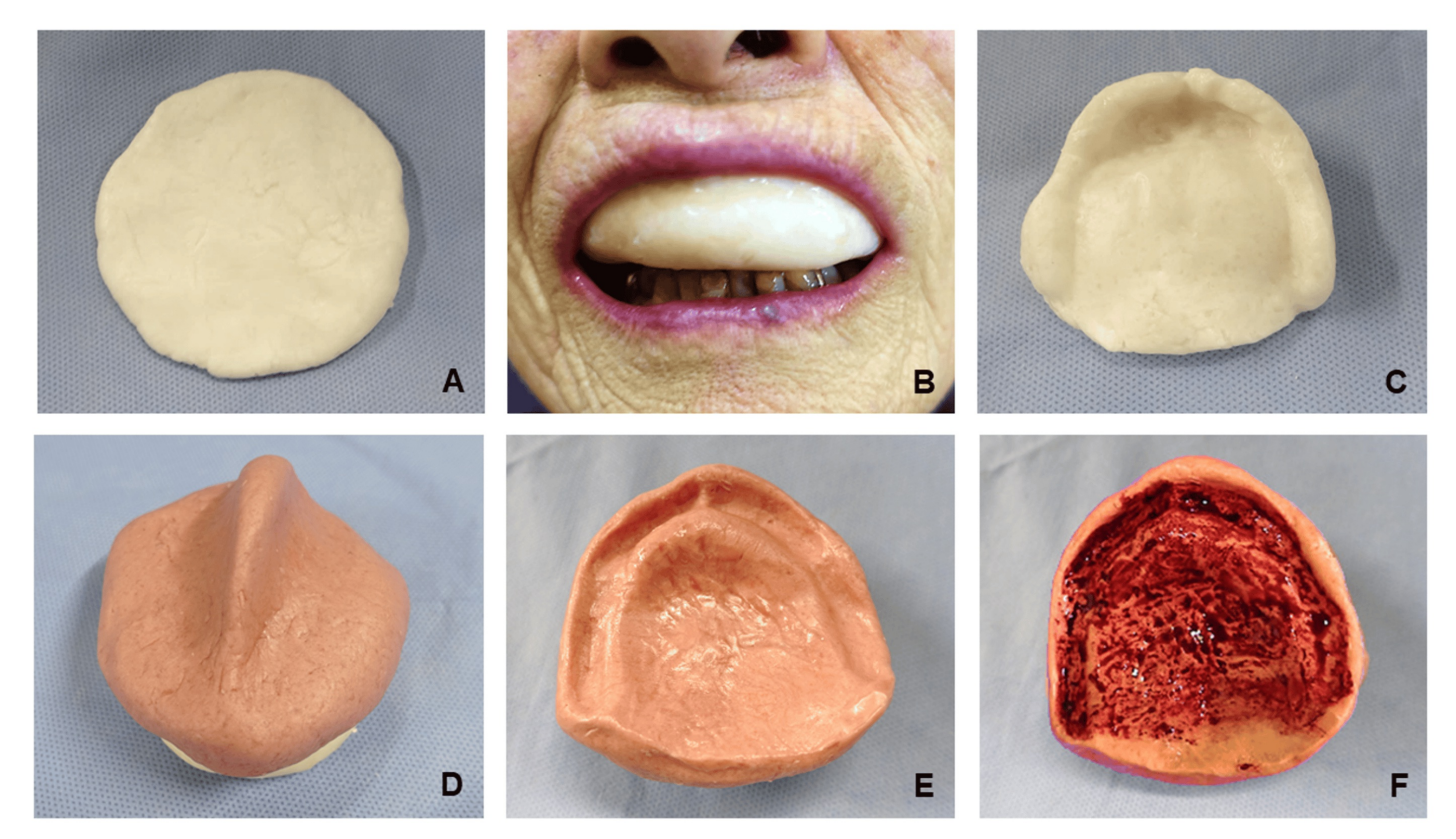
Beeswax for impression taking. A piece of beeswax for impression taking (A). Beeswax in the mouth of the subject (B). Negative impression (C). Beeswax pressed on the negative impression (D). Positive impression (working model) (E). Working model painted with red food coloring (F).

Japanese beeswax (Yamakei Co., Ltd., Osaka, Japan) was formed into a disc approximately 8 mm thick and 7 cm in diameter (Fig. 1A). The wax was softened in warm water and inserted into the oral cavity of the patient (Fig. 1B). An impression of the alveolar ridge and occlusal registration were obtained and chilled in cold water to form a negative mold (Fig. 1B-1C). Beeswax mixed with Japanese red food coloring (Benisei Co., Ltd., Tokyo, Japan) of Aspergillus mold (*Monascus purpreus*) was poured into the negative mold (Fig. 1D) to produce a positive mold (working model; Fig. 1E). The working model was painted with red food coloring to adjust the denture base (Fig. 1F).

**Figure 2:**
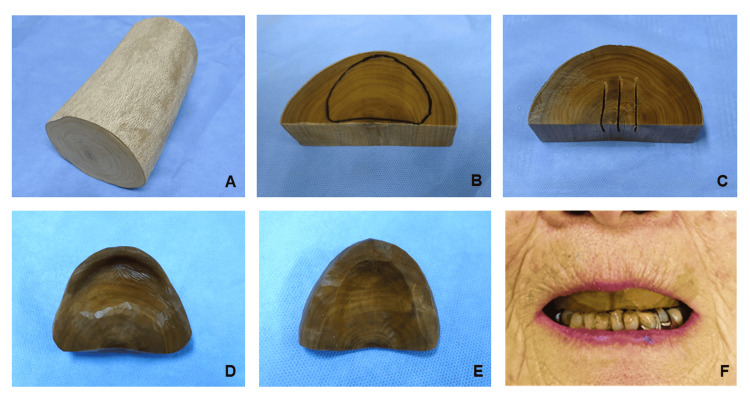
Materials for denture base. A log of boxwood (A). Boxwood after being cut, boiled, and stored in water (B). Preparation of the denture base and back side notched with a saw (C). Mucosal surface view (D) and occlusal view (E) of the denture base. Denture base in the mouth (F).

A log of boxwood (*Buxus microphylla var. japonica*) from Kagoshima (Fig. 2A) was cut lengthwise, and slices with a thickness of approximately 4 cm each were cut. The wood was boiled in a pot for 24 hours then stored in water for a month (Fig. 2B). The outer layer of the preserved boxwood became the area of the anterior teeth, and the center of the tree rings became the posterior border of the denture base (Fig. 2B). The back of the base was roughened using a saw to prevent cracking (Fig. 2C). Carving of the denture base began in the mucosal area (Fig. 2D).

**Figure 3:**
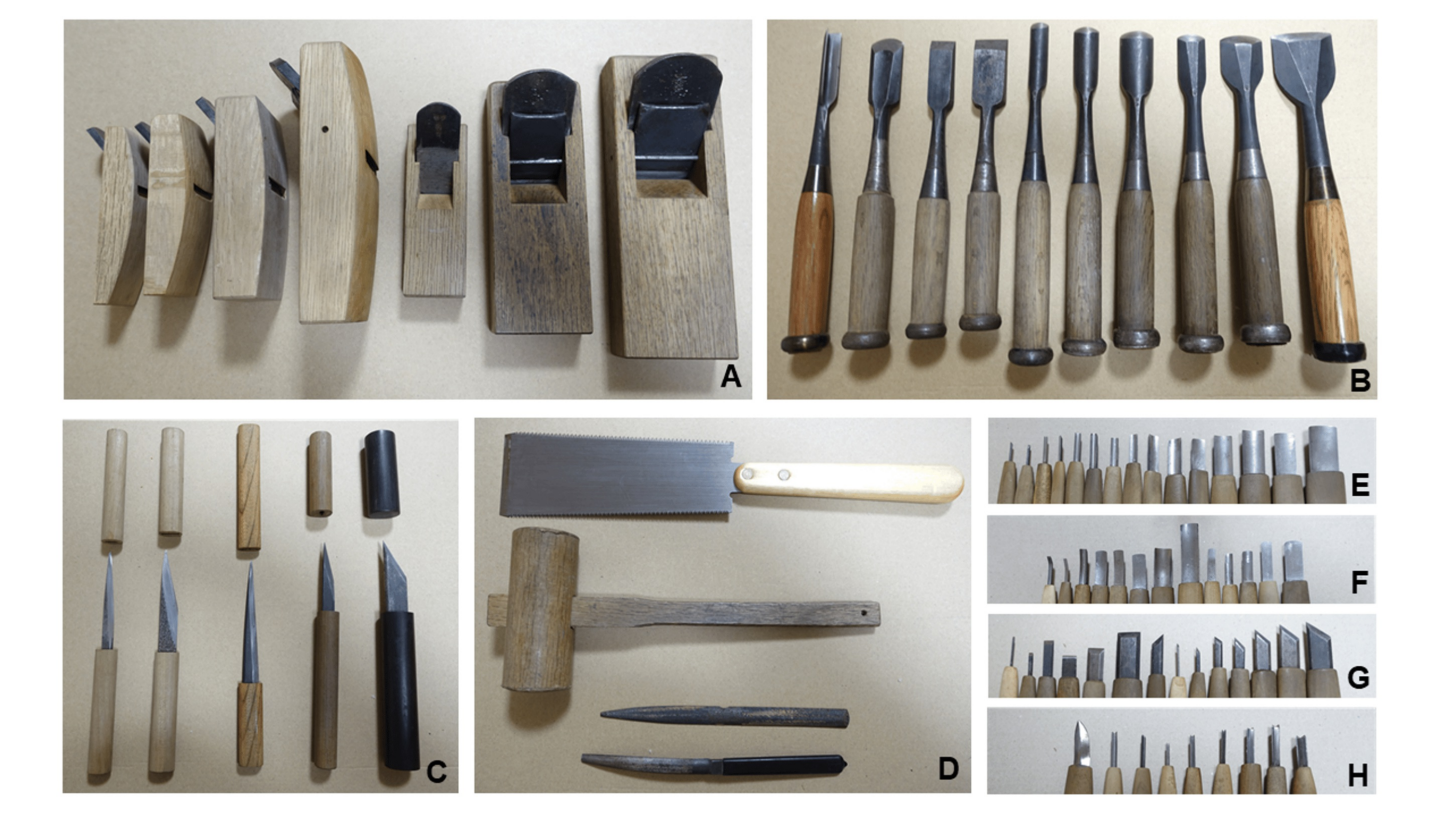
Tools for making the wooden plate denture. Planes (A), chisels (B), small knives (C), saw, wooden mallet, and files (D), and carving knives [round knives (E), curved round knives (F), flat and seal knives (G), and triangular knives (H)].

The tools used to fabricate the wooden plate denture are shown in Fig. 3A-3H. The surface of the denture in contact with the mucosa was first roughly carved using chisels and a hammer (Fig. 3B-3D). The base was subsequently carved using round chisels or carving knives while continually being checked by pressing it against the working model painted with red food coloring until the denture base fit perfectly. Finally, the base was finished using small carving knives (Fig. 3F-3H). After the base had satisfactorily adapted, the area from the outer gingival surface to the incisal edges of the teeth was cut using a saw or chisel. The shape of the palatal surface was then completed to an appropriate thickness (Fig. 2E) using round chisels (Fig. 3B). The adaptation of the completed denture base was checked in the mouth of the patient (Fig. 2F). Red food coloring mixed with flour from Hokkaido and water was applied to the gingiva, and the areas of the denture to which it was transferred were adjusted using a narrow round chisel or carving knife (Fig. 3F-3H).

**Figure 4:**
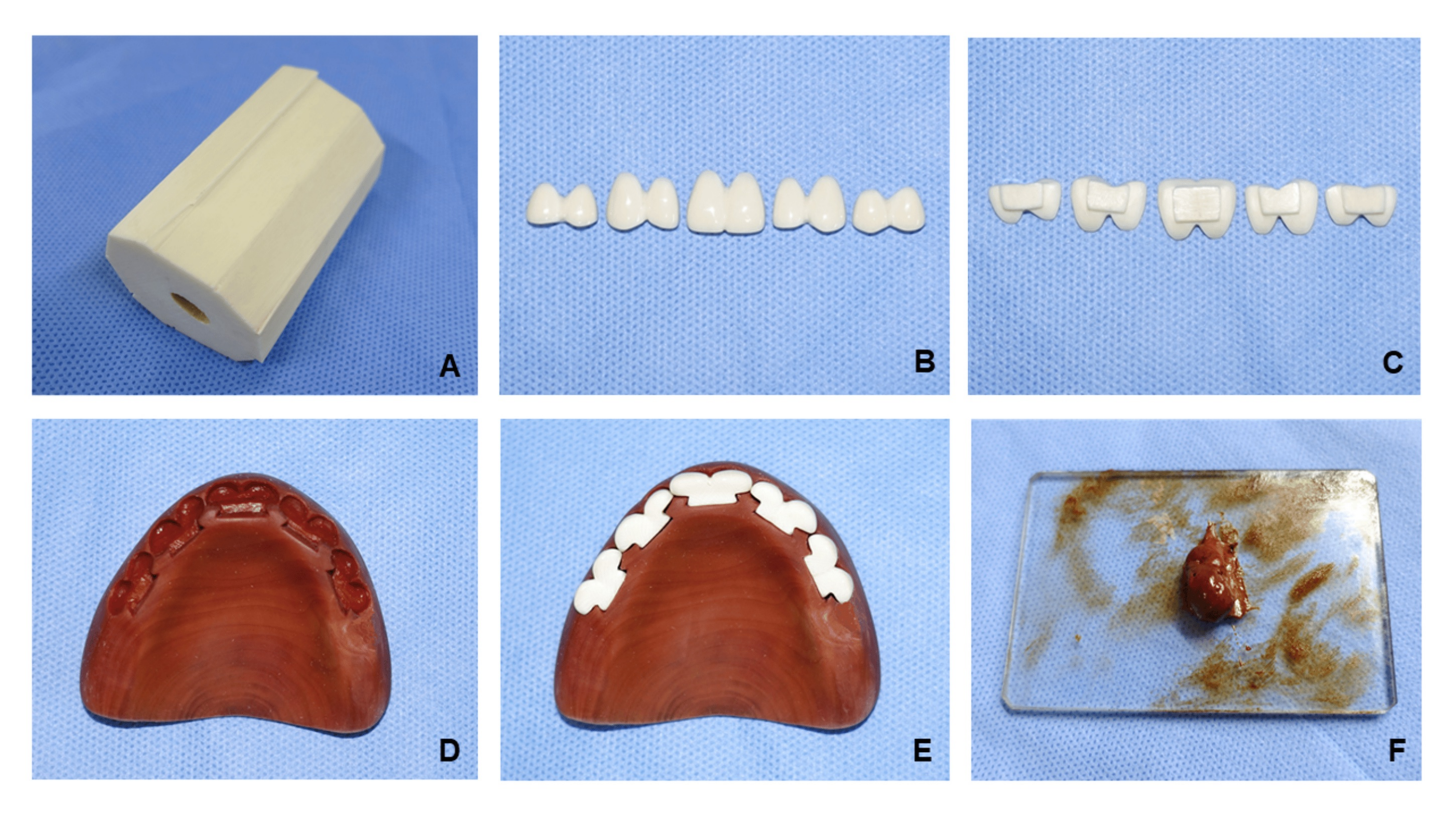
Materials for artificial teeth. Ivory for artificial teeth (A). The labial side (B) and palatal side (C) of artificial teeth formed a dovetail to fix the denture base. The denture base formed a dovetail (D). Teeth were glued with mugi-urushi (E) to the denture base (F).

When the denture was adequately adapted, the artificial anterior teeth up to the second premolars were carved from the ivory (Fig. 4A) of African elephants (*Loxodonta cyclotis*) (Motohashi Zougeten, Tokyo, Japan) using small knives, carving knives, and files (Fig. 3C-3H), while observing the occlusal relationship. The purchase of ivory was permitted in accordance with the Convention on International Trade in Endangered Species of Wild Fauna and Flora and with Japanese law. The ivory was carved into artificial teeth using carving knives and files and adapted to holes carved in a dovetail form into the denture base (Fig. 4B-4C). Only the facial surfaces of the premolars were carved for aesthetic reasons; the occlusal surfaces were not carved anatomically (Fig. 4B-4C). The denture base was carved such that the dovetail-shaped artificial teeth fit into it (Fig. 4D-4E). "Urushi" (Japanese raw lacquer) (Matsuzawa Urushi Kobo, Iwate, Japan) was mixed with wheat flour from Hokkaido kneaded with water, which is called "mugi-urushi" and creates a strong bond (Fig. 4F). The artificial ivory teeth were fixed with mugi-urushi to the base of the palatal surface (Fig. 4F). The mugi-urushi was allowed to harden completely over two to three weeks.

Red food coloring was applied to the positive mold and pressed against the wood, and the marked area was carved using carving knives until the base was completely fitted. Subsequently, red food coloring was applied to the mucous membrane of the alveolar ridge of the patient, the denture was fitted in the mouth, and the reddish portion on the inner surface of the denture was adjusted with chisels or carving knives until the fit was satisfactory.

**Figure 5:**
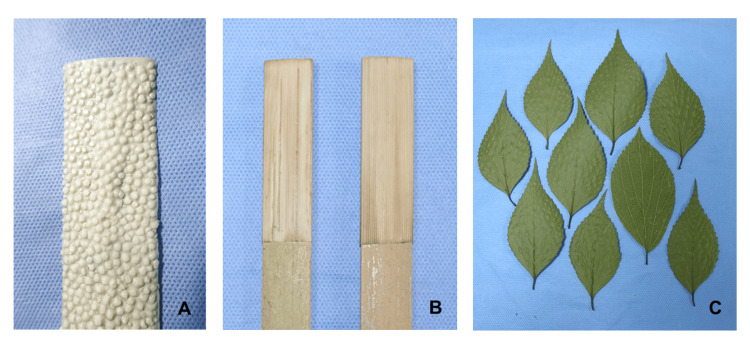
Materials used for polishing. Shark skin (A) and dried tokusa (scouring rush; *Equisetum hyemale*) (B) were cut to appropriate sizes and glued onto pieces of wood. The leaves of the muku tree (*Aphananthe aspera*) (C) were dried and used for final polishing.

Rough polishing was performed with shark skin (Fig. 5A) followed by tokusa (scouring rush; *Equisetum hyemale*) (Fig. 5B). Finally, the leaves of the muku tree (*Aphananthe aspera*) were used for the final polishing (Fig. 5C) [6].

**Figure 6:**
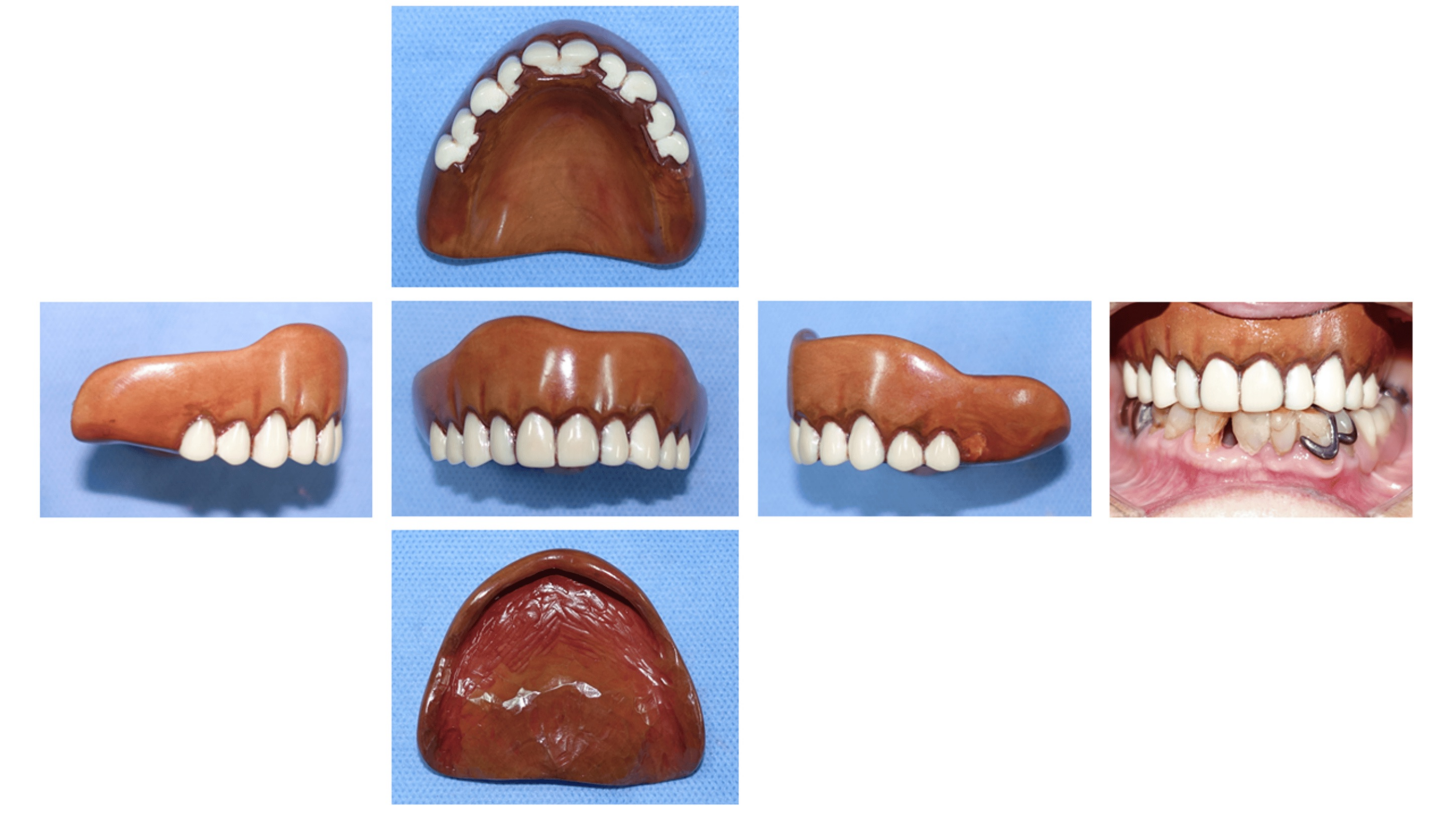
Completed wooden plate denture in place.

The wooden denture was completed and tried in the mouth of the patient (Fig. 6).

Assessment of masticatory performance

**Figure 7:**
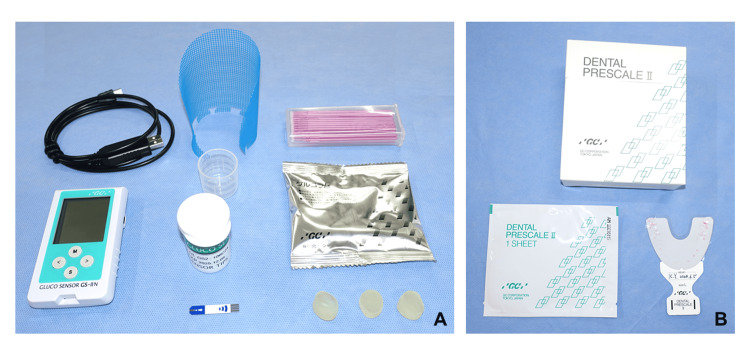
Assessment devices for masticatory performance. Masticatory performance was assessed using the Gluco Sensor-GS-II (A) and the occlusal force and masticatory ability were measured with the Dental Prescale II (B).

Masticatory performance was measured using the gummy-jelly test (Gluco Sensor-GS-II, GC; Tokyo, Japan) [7] (Fig. 7A). The patient was instructed to chew glucose-containing gummies (Glucolam) for 20 seconds on the main chewing side, being careful not to swallow saliva. Subsequently, 10 mL of water was lightly rinsed out of the mouth, the chewed gummies were spat out with water onto a filtration mesh, and the filtrate was analyzed for glucose elution using the Gluco Sensor-GS-II (GC; Tokyo, Japan). The maximum bite force was measured on the bilateral first molars on three occasions using an occlusal force meter (GM10, Nagano Keiki Co., Ltd., Tokyo, Japan) [8]. In addition, the average bite force was measured using the Dental Prescale II (GC; Tokyo, Japan) [9] (Fig. 7B). A pressure-sensitive sheet was inserted into the mouth, and the patient was instructed to clench in the intercuspal position for three seconds. After the test, the bite force on the pressure-sensitive sheet was analyzed using a bite force analyzer. The masticatory ability and maximal occlusal force were measured with both the currently worn dentures and wooden plate dentures.

Results

The complete wooden plate denture is shown in Fig. 6. The denture weighed 19.6 g.

The masticatory performance assessed using the Gluco Sensor-GS-II was 209 mg/dL with the current denture and 185 mg/dL with the wooden denture. The maximal occlusal force measured using the occlusal force meter was 14 N on the left and 18.2 N on the right with the current denture; 14.5 N on the left and 17.8 N on the right with the wooden denture. In addition, the mean occlusal force measured using the Dental Prescale II was 31.4 N (mean pressure: 26.4 MPa, maximal pressure: 41.2 MPa) with the current denture and 31.8 N (mean pressure: 31.8 MPa, maximal pressure: 49.4 MPa) with the wooden denture. The patient reported no pain or other problems during the examination and was able to chew the test food and clench adequately.

## Discussion

To our knowledge, this is the first trial to regenerate the characteristic Japanese wooden plate denture using the same materials, tools, and techniques used 400 years ago. A complete maxillary denture was fabricated and showed a satisfactory masticatory performance. As shown in this study, the wooden denture may be functionally comparable to modern complete dentures.

**Figure 8:**
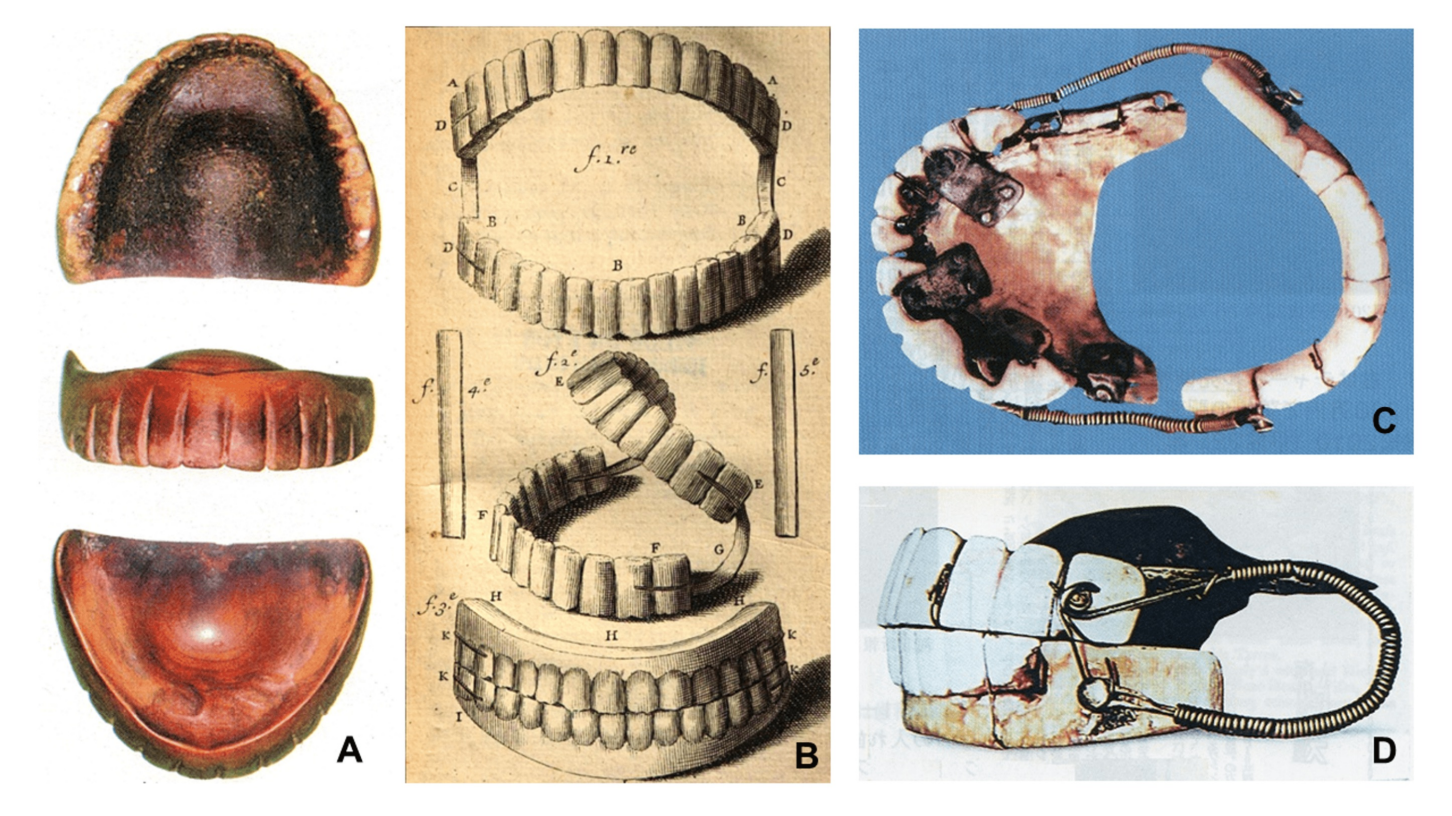
Complete dentures used from the 16th to the 18th centuries. The form of the oldest complete denture in the world, used in the 1530s (A) [10], is the same as that of modern dentures, and the retention of dentures by suction force is identical to that in modern theory. Complete dentures with leaf metal springs by Pierre Fauchard in 1728 (B) [12], and those used by George Washington from the 1770s (C, D). Because of the elastic spring (B), the wearer could not close the mouth without clenching and could not speak [3]. NOTE: Panel B, public domain image. Panels A and C, reproduced with permission from the original authors.

Wooden plate dentures are unique to Japan and are not found in other countries as they originate from skilled Japanese craftsmanship [1-3]. Although records exist stating that wooden dentures were made in the 13th century [1, 2], the oldest denture discovered is the maxillary complete denture (Fig. 8A), which was worn by Nakaoka Tei, who died in 1538 [10] (please note that in this text, Japanese names are written in the order of surname and first name). Nakaoka Tei was a priestess who founded a temple. After her death, her hair and dentures were carefully stored at the temple as treasures [10]. The denture is identical in form to modern dentures, and the retention by suction force is the same as that in modern theory. At the time, craftsmen probably understood from long-standing trial and error that maxillary complete dentures were influenced by the intervention of saliva, creating a negative pressure and causing the denture to adhere to the alveolar ridge [3]. In that regard, the dentist of King Louis XVI in France refused a patient who had requested a complete denture in 1737, stating the following: "Making dentures in a mouth without a single tooth left and without all the conditions to connect it is as difficult as attempting to build a building in the air [11]." In 1728, Pierre Fauchard (1679-1761), who is often referred to as the founder of modern dentistry, reported the development of maxillary and mandibular dentures (Fig. 8B) [12]. Metal springs were installed at the distal end of the molars of the upper and lower dentures, and the elasticity of the spring maintained the fall of the maxillary denture (Fig. 8B). George Washington (1732-1799) also used similar dentures (Fig. 8C-8D); however, mastication with these dentures was impossible. The dentures were inserted for aesthetic purposes to make missing teeth less noticeable [3]. Two hundred years earlier than the “founder of modern dentistry,” unknown Japanese craftsmen had applied far superior techniques and theories [3]. In Europe and the United States, not until the 19th century did James Gardette notice that upper full dentures could be maintained using suction under negative pressure [13]. The clinical use of dentures using suction retention began in the United States after Charles Goodyear invented vulcanite dentures in 1855 [14].

Gilded-bronze Buddhist statues are generally made by wax casting using beeswax. This beeswax technique was presumably later applied to create denture impressions [1]. Western literature on the history of dentistry [13, 14] reports that Matthias Gottfried Purmann first made an impression using beeswax in 1711; nevertheless, this technique was already commonly used in Japan by unknown craftsmen more than 200 years prior [3]. Supposedly, skilled craftsmen such as Buddhist sculptors, Noh mask sculptors, and netsuke carvers favorably made wooden dentures for their relatives and acquaintances; they may have subsequently switched jobs and become denturists [1-3].

Although the technique of fabricating wooden plate dentures is secret and is not documented because it was passed down verbally in the apprenticeship system, several authors have described these methods since then [1-3]. In a review article examining 145 complete and partial wooden plate dentures, the denture base material was boxwood (*Buxus microphylla var. japonica*) (80%), plum (*Prunus mume*) (4.1%), ivory (0.7%), and black persimmon (*Diospyros kaki Thunb.*) (0.7%) [3]. Boxwood is very dense and strong and is used for stamps and combs in Japan.

Three methods were used to fabricate artificial anterior teeth and premolars. The simplest denture was made from the same wood as the base and was similar to the oldest denture (Fig. 8A). After the Edo period, other materials were incorporated into dentures. Artificial teeth were made from the same wood as the base, such as boxwood (34.7%), pagodite (29.2%), animal bone (12.5%), extracted teeth (6.5%), ivory (4.2%), whale teeth (3.5%), other wood (2.1%), ebony (1.4%), and animal horn (1.4%) [3]. Ebony and black persimmon were particularly used to create artificial teeth for women to imitate the tradition of "ohaguro" (blackened teeth), which has existed in Japan since ancient times and was seen as a custom among married women [3]. The number of artificial teeth was eight teeth in 51%, 10 teeth in 13.8%, and six teeth in 2.3% [3]. The anterior teeth to the premolars accounted for the majority of aesthetic improvement. Mesiodistal holes were made on the sides using a potter's wheel and ligated with a shamisen string or glued with Japanese lacquer. In this study, artificial teeth carved with ivory were embedded in the base in a dovetail shape using mugi-urushi (Fig. 5F). At the time, wooden dentures were very expensive and could only be worn by wealthy people. Dentures with the anterior teeth made from a single piece of denture base material were the least expensive. Artificial teeth made of pagodite or bovine bone were inexpensive and often used. Extracted natural teeth were the most expensive. Ivory was not used often because it was precious and expensive.

One drawback of using wood is its deformation upon exposure to moisture. The purpose of the boiling step is to remove the resin from the wood. In addition, because wood expands slightly when the humidity is high, storage in water may prevent deformation, even in the moist state of the oral cavity. When a wooden plate denture was first inserted, two or three sheets of Japanese paper ("kizuki-gami," unprocessed paper purely made from paper mulberry trees) were stacked and attached to the inner surface of the denture then peeled off one by one while observing the progress [5]. More than half of the inner surfaces of wooden dentures have chisel marks [5]. Some researchers believe that small indentations on these chisel marks provide negative pressure and contribute to denture maintenance. Rice grains were kneaded into a paste on the inner surface of the denture and used as a denture stabilizer [3]. After a long period of denture use, a gap would inevitably occur between the inner surface of the denture and the mucous membrane of the alveolar ridge. Denture adhesives, made by kneading rice grains into a paste, were safe and easy to obtain [3].

Various methods are clinically applied to assess the masticatory ability of patients wearing full dentures. Diverse methods have been developed and tested, and they have been classified as direct or indirect [15]. Direct methods include the sieve method using peanuts [16] or measuring the amount of glucose extracted from chewing gummy jelly [7, 17]. The position paper from the Japanese Society of Gerodontology recommends the latter method [18]. Indirect methods include the measurement of occlusal force, mandibular movement, or electromyography of masticatory muscles. Occlusal force can be assessed for each molar on one side using an occlusal force meter [16], and mean bite force can be measured using the Dental Prescale [17]. Our patient was satisfied with her current dentures and felt no inconvenience, and her masticatory performance maintained sufficient values. Shiga et al. [17] reported that the standard amount of glucose extracted for denture wearers was 100 mg/dl. The masticatory performance measured using the Gluco Sensor-GS-II in our study was 209 mg/dL with the current denture and slightly reduced to 185 mg/dL with the wooden denture. Assessments are typically performed after a certain habituation period. Shiga et al. suggested that masticatory performance should be assessed in denture wearers two months after denture insertion [17]. In this study, the assessment was conducted just after the trial of the wooden denture. The masticatory ability would improve if the subject were re-examined after having worn the wooden denture for two months, but this would be difficult from an ethical standpoint. Notably, the occlusal force of the patient had declined considerably. The maximal occlusal force measured using the occlusal force meter was 14 N on the left and 18.2 N on the right with the current denture, 14.5 N on the left and 18.4 N on the right with the wooden denture. In addition, the mean occlusal force measured using the Dental Prescale II was 31.4 N (mean pressure: 26.4 MPa, maximal pressure: 41.2 MPa) with the current denture and slightly increased to 31.8 N (mean pressure: 31.8 MPa, maximal pressure: 49.4 MPa) with the wooden denture. These results suggest that wooden dentures can restore masticatory ability to the same extent as current dentures.

The author fabricated a wooden plate denture and was able to reproduce this practice for the first time. However, it falls significantly short of the exquisite wooden dentures crafted during the Edo period, both in terms of aesthetics and likely in functionality as well [1-3]. The technical procedures required a long time, and the task of arranging the dovetailed ivory teeth on the denture base was particularly difficult. Carving in three dimensions without using an articulator was necessary, and the denture had to be remade if too much was removed. The skilled denturists of the time likely employed their exceptional techniques and craftsmanship to create wooden dentures that were adequate for chewing. During the Edo period, Japan developed a highly sophisticated culture and craftsmanship unique to the country [3, 19]. Surprisingly, nameless craftsmen without any knowledge of dentistry and local anatomy thought of constructing wooden dentures based only on their advanced techniques, observations, and trial and error. The fabrication of wooden plate dentures attained nearly perfect forms by the mid-to-late 16th century [1-3]. In the late Edo period, Sato reported that elastic and hard foods such as octopus could be chewed using wooden dentures [20]. Wooden plate dentures with attrition and tartar would demonstrate sufficient masticatory ability [3]. During the Edo period, specialized and highly skilled denturists likely crafted far superior dentures with relative ease compared to that produced in this study.

## Conclusions

Japan was the first country in the world to use complete dentures that retain suction under negative pressure in the 16th century. A characteristic Japanese wooden plate denture was reproduced using materials and methods from the Edo period and showed satisfactory masticatory performance. Although Japanese wooden dentures have not been mentioned in Western literature and are unknown, they hold significant importance in the evolution of denture technology. Although staying attuned to the latest technological advancements is essential, we must also recognize the value of historical knowledge and draw inspiration from the wisdom of the past.
